# Evaluation of microarray-based DNA methylation measurement using technical replicates: the Atherosclerosis Risk In Communities (ARIC) Study

**DOI:** 10.1186/1471-2105-15-312

**Published:** 2014-09-19

**Authors:** Maitreyee Bose, Chong Wu, James S Pankow, Ellen W Demerath, Jan Bressler, Myriam Fornage, Megan L Grove, Thomas H Mosley, Chindo Hicks, Kari North, Wen Hong Kao, Yu Zhang, Eric Boerwinkle, Weihua Guan

**Affiliations:** Division of Biostatistics, School of Public Health, University of Minnesota, Minneapolis, MN 55455 USA; Division of Epidemiology & Community Health, School of Public Health, University of Minnesota, Minneapolis, MN 55455 USA; Human Genetics Center, School of Public Health, University of Texas Health Science Center at Houston, Houston, TX 77030 USA; Department of Neurology and Department of Medicine, University of Mississippi, Jackson, MS 39216 USA; Department of Epidemiology, School of Public Health, University of North Carolina, Chapel Hill, NC 27516 USA; Department of Epidemiology, Johns Hopkins Bloomberg School of Public Health, Baltimore, MD 21205 USA; Department of Computer Science, Saint John’s University, Collegeville, MN 56321 USA

**Keywords:** DNA methylation, Infinium 450 K chip, Technical error, Intraclass correlation, Normal mixture models

## Abstract

**Background:**

DNA methylation is a widely studied epigenetic phenomenon; alterations in methylation patterns influence human phenotypes and risk of disease. As part of the Atherosclerosis Risk in Communities (ARIC) study, the Illumina Infinium HumanMethylation450 (HM450) BeadChip was used to measure DNA methylation in peripheral blood obtained from ~3000 African American study participants. Over 480,000 cytosine-guanine (CpG) dinucleotide sites were surveyed on the HM450 BeadChip. To evaluate the impact of technical variation, 265 technical replicates from 130 participants were included in the study.

**Results:**

For each CpG site, we calculated the intraclass correlation coefficient (ICC) to compare variation of methylation levels within- and between-replicate pairs, ranging between 0 and 1. We modeled the distribution of ICC as a mixture of censored or truncated normal and normal distributions using an EM algorithm. The CpG sites were clustered into low- and high-reliability groups, according to the calculated posterior probabilities. We also demonstrated the performance of this clustering when applied to a study of association between methylation levels and smoking status of individuals. For the CpG sites showing genome-wide significant association with smoking status, most (~96%) were seen from sites in the high reliability cluster.

**Conclusions:**

We suggest that CpG sites with low ICC may be excluded from subsequent association analyses, or extra caution needs to be taken for associations at such sites.

**Electronic supplementary material:**

The online version of this article (doi:10.1186/1471-2105-15-312) contains supplementary material, which is available to authorized users.

## Background

DNA methylation is one of the most commonly occurring epigenetic phenomena in the human genome. It is one of the major regulators of gene transcription and plays a vital role in many cellular processes. In the last decade, numerous studies have shown that abnormal methylation patterns are linked to phenotypic differences and development of disease [1–6].

Recent technological advances have provided multiple platforms for systematically interrogating DNA methylation variation across the genome [7]. Among them, the Illumina HumanMethylation450 BeadChip (HM450) (Illumina, Inc.) is a new-generation array constituting a major extension of the previous Infinium HumanMethylation27 BeadChip (HM27) (Illumina, Inc.) and can be used to assess the methylation condition of more than 480,000 cytosines distributed over the entire genome. Sandoval et al. [8] validated the HM450 chip by showing that methylation patterns measured in colorectal cancer cell lines and normal mucosa were consistent with those found in bisulfite genomic sequencing. Other recent studies have extensively evaluated data generated from this chip and developed data processing and analysis pipelines [9–11].

Similar to other microarray experiments (e.g. RNA expression), it is important to evaluate the impact of technical variation in the measurement. A well-known source of bias for epigenome-wide association studies (EWASs) of DNA methylation is the so-called “batch effect”, which is largely caused by technical differences from one chip to the next. Although many statistical methods have been proposed to correct for batch effects [12–17], no approach has been generally accepted. Alternatively, it will be useful to consider statistical measures that can quantify the extent to which the measured methylation level at a specific CpG site is affected by technical errors. For CpG sites with large inter-individual variation, it may be reasonable to assume that technical differences will have relatively low impact at these sites. However, the HM450 chip contains a large number of CpG sites with little inter-individual variation in methylation levels, for which it is critical to consider additional statistics to evaluate the impact of technical errors.

In experiments, technical replicates are often included which can be used to evaluate the consistency of measurement. Meng et al. [18] suggested using technical replicates to identify and exclude “non-variable” CpG sites, at which the technical “noise” outperforms true biological variation. Their data came from the Illumina GoldenGate methylation array and consisted of 311 samples assayed at 1,505 sites. However, no comprehensive work has been done for the HM450 chip.

In the Atherosclerosis Risk in Communities (ARIC) study, the HM450 chip was used for genome wide profiling of DNA methylation in 2,873 African-American individuals assayed at 485,577 CpG sites, with 130 replicate pairs or triplets included. We evaluated the consistency of methylation measurement at each CpG site based on the intraclass correlation coefficient (ICC), which compares the within- and between-replicate variations. We next modeled the ICC values using a mixture model approach to classify CpG sites on the HM450 chip into high and low reliability clusters. We demonstrated the relationship of this classification to the results of an association study between methylation level and smoking status of ARIC study participants.

The aim of this work was to apply a statistical procedure to identify CpG sites which give consistent values for methylation levels in DNA extracted from peripheral blood using the HM450 chip (or a similar methylation profiling array), thereby providing insight into the performance of this chip across CpG sites. We hope that our results will facilitate subsequent EWAS analyses.

## Results

Based on the ICC, we quantified the reliability of methylation measurements on the HM450 chip using technical replicates included in the analysis of DNA methylation in ARIC study participants. The CpG sites were then classified into low and high reliability groups by modeling the distribution of the ICC. We further demonstrated the relationship of measurement reliability to the associations between methylation and smoking status in the ARIC study.

### Distribution of ICC

**Figure 1:**
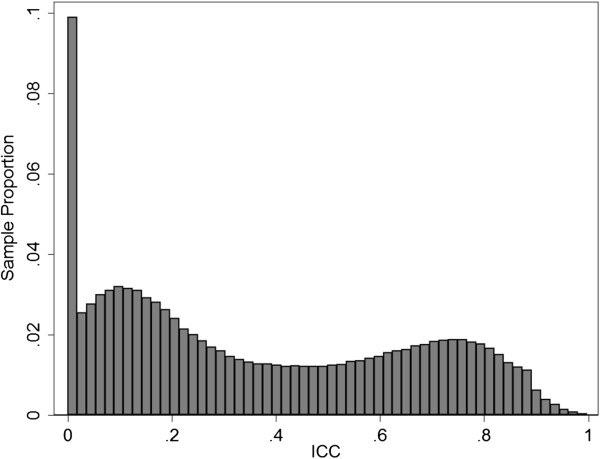
**Distribution of intraclass correlation coefficients (ICC) in the ARIC methylation data.**

Using the 265 technical replicates available in the ARIC methylation data, we calculated the ICC value for each CpG site assayed on the HM450 chip. After excluding sites with low pass rate, 473,788 CpG sites were analyzed. The distribution of ICC is shown in Figure 1. The median ICC value was 0.30. We observed one cluster of sites with relatively high ICC values (mode ~ 0.75), one cluster with relatively low ICC values (model ~ 0.10), and an additional cluster of sites with ICC of 0 (n = 36,017). This empirical distribution supported the truncated/censored normal mixture model we assumed.

**Figure 2:**
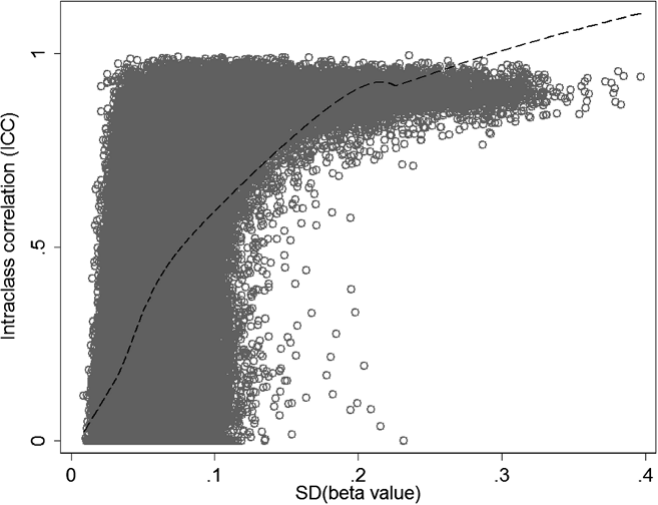
**Relationship between ICC and standard deviation of methylation level.** The dashed line is the fitted curve using a local regression (LOWESS) approach.

We further investigated how the ICC values change with the variation (standard deviation, SD) of methylation levels. It is reasonable to assume that when the SD is large, the relative impact of technical error becomes small and methylation measures for technical replicates will in general be consistent. Using a locally weighted scatterplot smoothing (LOWESS) approach, we observed a positive correlation between ICC and variation of methylation (Figure 2). In practice, an EWAS can apply a filter based on methylation variation to focus on CpG sites with large variation, e.g., SD > 0.1 [19]. In our results, among CpG sites with SD of 0.1 or above (n = 21,593), 78% had ICC > 0.80, and 95% had ICC > 0.37 (belonging to the high reliability cluster; see below). However, these sites accounted for only 4% of the CpG sites surveyed by the HM450 chip. It is therefore important to assess measurement reliability on the CpG sites with low or moderate variation in methylation levels.

**Figure 3:**
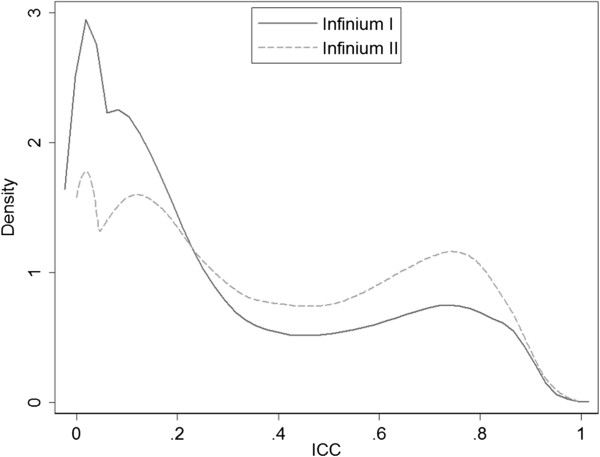
**Distribution of ICC values for the Infinium I and II probes.**

**Figure 4:**
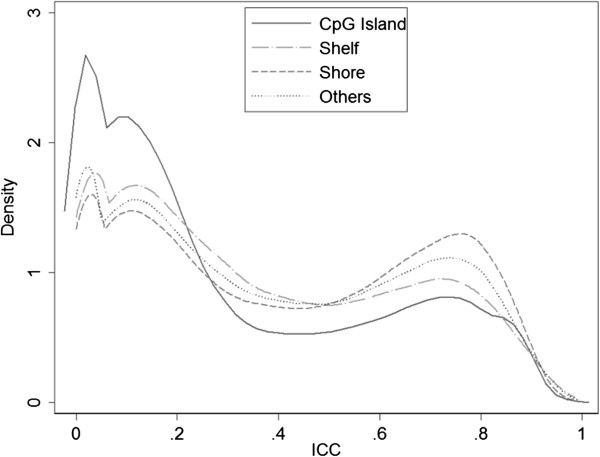
**Distribution of ICC values and their relation to CpG islands.**

We examined the distribution of ICC values by the two types of Infinium probes (Figure 3). Compared to the Infinium II probes, a larger proportion of Infinium I probes showed smaller ICC values and less consistency between technical replicates. Specifically, there are 31.7% Infinium I probes with ICC > 0.4, while 46.2% of Infinium II probes have ICC > 0.4. Similar comparison was done for CpG sites’ relation to CpG islands (Figure 4). The Infinium I probes have higher proportions in CpG islands than the Infinium II probes [20], and we observed less sites in CpG islands with high ICC compared to those not in CpG islands.

**Figure 5:**
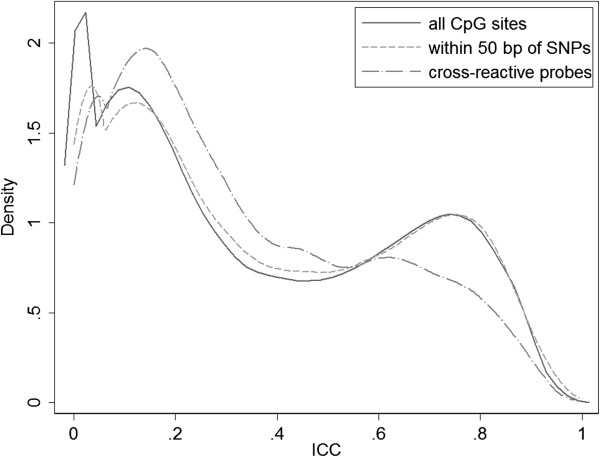
**Distribution of ICC for all CpG sites, CpG sites within 50 bp of SNPs, and cross-reactive probes reported by Chen et al.** [21] **.**

Chen et al. [21] reported a list of cross-reactive probes (~6%) on the HM450 chip that can co-hybridize to alternate sequences on the genome. We observed enrichment for these probes with low ICC values (Figure 5), suggesting that cross-reactivity may explain the low reliability at some CpG sites. In addition, we examined the distribution of ICC for CpG sites within 50 bp of SNPs [22], but found no significant difference from rest of the CpG sites (Figure 5).

### Classification of CpG sites using mixture models

To classify the CpG sites based on the consistency of methylation measures between technical replicates, we used a mixture model approach to the observed ICC values. We first fit a censored normal mixture model, which assumes a high reliability cluster of CpG sites (ICC following a normal distribution) and a low reliability cluster (ICC following a censored normal distribution). In addition, we fit a truncated normal mixture model to the non-zero ICC values, to allow flexibility in modeling the cluster of ICC equal to 0. Estimates of the mixing proportion of the high reliability component, means and standard deviations of the two normal distributions are given in Table 1.

**Table 1:** **Fitted models for ICC distribution**

	Censored normal mixture	Truncated normal mixture
*p*	0.55	0.67
*μ*_1_	0.15	0.04
*μ*_2_	0.67	0.72
*σ*_1_	0.14	0.28
*σ*_2_	0.14	0.11
Maximized log likelihood	-86500	-80230

**Figure 6:**
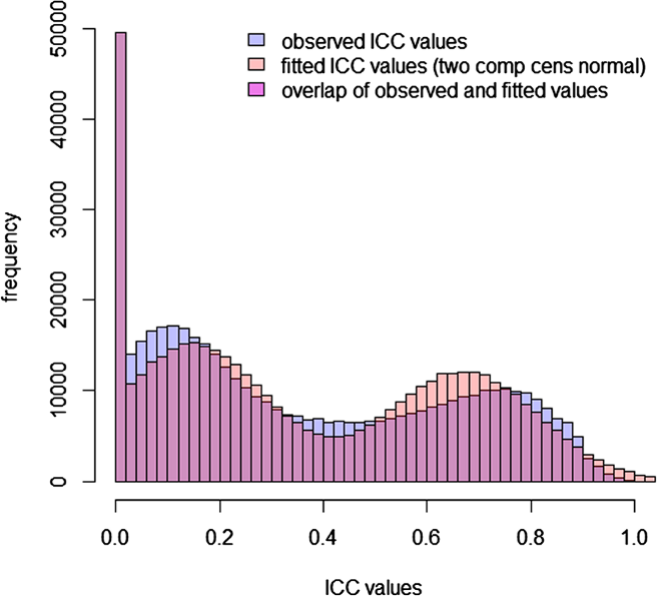
**Observed and fitted distributions of ICC values using the censored normal mixture model.**

**Figure 7:**
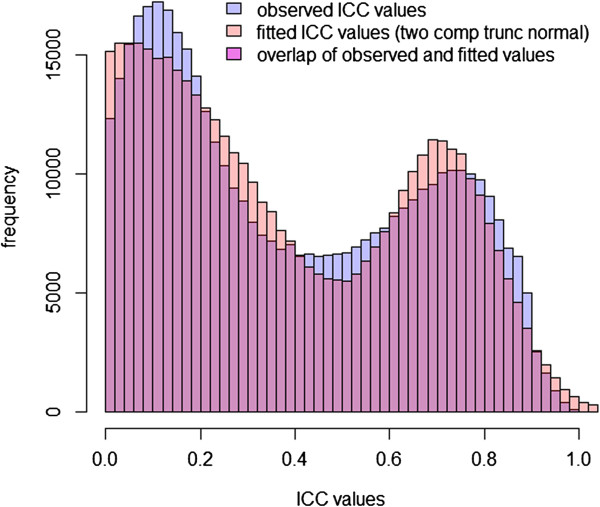
**Observed and fitted distributions of ICC values using the truncated normal mixture model (for non-zero ICC).**

The observed and fitted distributions of ICC values for the two models are shown in Figures 6 and 7. The maximized log likelihood value was compared for the two mixture models (Table 1). The truncated normal mixture model using sample proportions to estimate the relative size of the cluster for ICC = 0 had a higher maximized log likelihood than that of the censored normal mixture model. In the following discussion, we grouped the CpG sites with ICC = 0 as a separate cluster (“zero-ICC”), and classified the sites with non-zero ICC values into low reliability and high reliability clusters based on the posterior probability *π* from the fitted truncated normal mixture model. To maximize the size of the high reliability cluster, we chose a rather low cutoff value of 0.01 for *π* (see Discussion), which corresponds to an ICC value of 0.37. The numbers of CpG sites classified into each of the three clusters were: 36,017 (ICC = 0), 228,231 (low reliability cluster), and 209,540 (high reliability cluster).

### Data application: association with smoking status

Next, we investigated how association patterns between phenotypes and methylation levels vary across different reliability clusters using smoking status from the ARIC Study as an illustrative example. Complete information on methylation levels, smoking status, and covariates was available for 2,835 African American participants. The average age was 56.7 years (standard deviation, SD, 5.9 years), 63.4% were female, and 25.6% were current smokers. 874 CpG methylation sites displayed genome wide significant association (p < 10^-7^), with current smoking status, or 723 CpG sites after excluding those with at least one common SNP within 50 base pairs. Among the 723 associated sites, only 31 were from the low reliability cluster, and none from the zero-ICC cluster.

**Figure 8:**
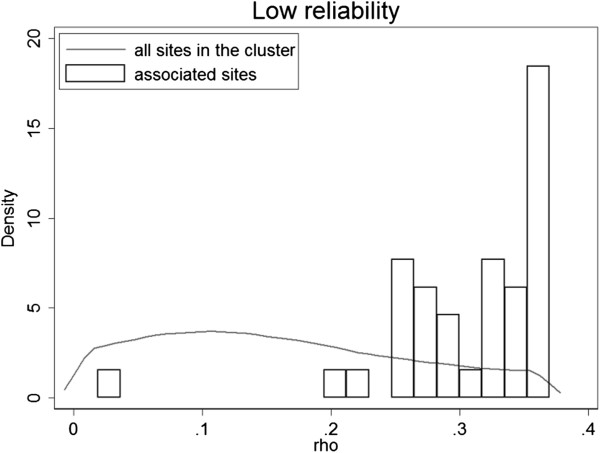
**Distribution of ICC values of smoking-associated CpG sites belonging to the low reliability cluster.**

**Figure 9:**
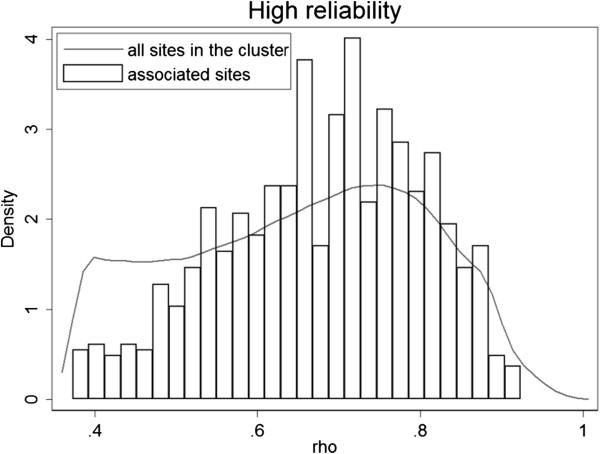
**Distribution ICC values of smoking-associated CpG sites belonging to the high reliability cluster.**

We further investigated distributions of the smoking-associated CpG sites in the low and high reliability clusters, and compared to the distribution of ICC values in each cluster (Figures 8 and 9). In both clusters the number of associated sites increases with increase in ICC values. In the low reliability cluster, most of the associated CpG sites had ICC > 0.2, while a high density of low ICC (<0.2) values was observed in this cluster. In the high reliability cluster, the distribution of associated sites was more proportional to the distribution of ICC. The relationship between association p-values and ICC at all CpG sites is shown in Additional file 1: Figure S1.

## Discussion

Recent technological advances have provided high-throughput array for systematically interrogating DNA methylation across the genome. This allows investigators to evaluate regions of the genome in which variation in DNA methylation may influence gene expression and ultimately disease risk. One of the potential problems that could threaten the validity of methylation-phenotype association results is the variability of technical measurements (e.g., batch effects). Here we used technical replicates to assess reliability of methylation measurement on CpG sites interrogated by the HM450 chip. Using a mixture model approach, we classified CpG sites into low and high reliability groups. The ICC value can serve as a measure to quantify the impact of technical errors, especially batch effects. In addition, CpG sites with low reliability could be excluded in subsequent analyses to improve the power of EWASs by reducing the burden of multiple hypothesis tests.

In our analysis of 265 technical replicates available as part of the ARIC methylation data, we classified CpG sites on the HM450 chip by a mixture of truncated normal and normal distributions, with ~44% sites in the cluster of high reliability. For CpG sites with high ICC values, the variation between independent biological samples is relatively large compared to the variation due to technical error. We expect statistical tests performed for the sites with high ICC will have more power to detect association between methylation level and phenotype of interest than for the sites with low ICC. In the follow-up EWAS of smoking status, we showed that a majority (96%) of genome-wide significant associations are from the high reliability cluster, even though only 44% of all sites were in this cluster.

The ICC provides a measure of reliability for the methylation measurement. In EWASs, it is well known that batch effects can threaten the validity of association results [14]. Commonly used methods for batch effects correction include empirical Bayes methods [17], surrogate variables [15, 16], and linear mixed-effects (LMM) models. However, it is difficult to completely control or remove batch effects in association tests. Alternatively, we can use ICC to quantify to what extent the batch effects can affect the methylation measures. The probes for CpG sites with low ICC are expected to be more vulnerable to technical variability including batch effects. Similar to the ICC for replicates, we calculated ICC for chip effects (ICC_chip_), which is the proportion of variation in measured methylation levels due to chip difference in the experiment. Additional file 1: Figure S2 shows that when ICC_chip_ is close to 1, most of the variation in methylation measures is due to technical “errors”. Reliability of methylation measures is therefore low at these CpG sites, with ICC for replicates close to 0. On the other hand, when ICC for replicates is close to 1, technical variability has minimal contribution to the methylation measures, and little batch effects are observed (ICC_chip_ ≈ 0).

In addition to the EWAS of smoking status using LMM, we performed the EWAS using a linear regression model without adjusting for chip and chip position (results not shown). There were 1,783 CpG sites significantly associated with smoking status, after excluding sites with low pass rate or with at least one common SNP within 50 base pairs. Among these sites, there was no site in the zero-ICC cluster, 253 sites from the low reliability cluster, and 1,530 sites from the high reliability cluster. All except 9 sites identified by the LMM were also genome-wide significant in this model. The number of significant sites without adjustment for chip and chip position was approximately 2.5-fold higher than we observed in the EWAS with those adjustments. We hypothesize that some of the additional associations are false positives due to technical errors. We also compared our results to published EWASs of smoking studies [23–27], which were carried out in individuals of European and African ancestry. We were able to validate ~80% of the reported signals with genome-wide significance. Of these, 32 sites were found within the body of the aryl-hydrocarbon receptor repressor (AHRR) gene, with a 15% decrease in the mean methylation level of cp05575921 in current smokers compared to non-smokers (p = 6.2 × 10^-196^) as previously shown in the other studies.

The ICC value is determined by two variance components,  for variance between independent samples and  for variance within replicates of the same sample. When sample characteristics (e.g., cell type composition, sample ethnicity, or genetic variants) are controlled for,  becomes smaller which leads to smaller ICC than the current estimates (in Additional file 1: Figure S3). When batch effects are controlled for, both  and  can be reduced and the ICC can increase or decrease at different CpG sites (in Additional file 1: Figure S4).

The empirical distribution of C we observed is similar to that of Meng et al. [15], in which an earlier version of the Illumina GoldenGate array having 1,505 CpG sites was used. In their analysis, Pearson product–moment correlation was primarily used between replicate pairs, although ICC was also introduced. Our analysis updates the information for the HM450 chip, and focuses on ICC which allows more than two replicates per biological sample.

Meng et al. observed that, compared to CpG sites not in CpG islands, a higher proportion of sites in CpG islands showed low inter-individual variation in methylation levels relative to within-individual variation (“non-variable”), consistent with a previous finding by Bock et al. [28]. Our results further confirmed this observation. In contrast, Dedeurwaerder et al. [10] studied technical replicates of a human colon cancer cell line and showed that the probe-wise variation of methylation levels were greater for Infinium II probes than those for Infinium I probes. However, their findings were limited to within-individual variation, with no comparison to inter-individual variation. In addition, given the small number of replicates, the impact of batch effects was minimal in their study. Therefore the results were not directly comparable.

From the distribution of ICC values we noted that the distribution of high ICC component is slightly left skewed, with few CpG sites having ICC close to 1. Because of the normal distribution assumption for this component in the mixture model, this skewness may lead to an underestimate for the corresponding variance parameter  and under-fit the distribution of ICC in the middle (0.2-0.5), in both of the truncated and censored normal mixture models. The direct consequence is that CpG sites with moderate ICC values will have a small posterior probability of being in the high reliability cluster. As a possible remedy, we considered three-component mixture models, with an additional normal distribution component to model the moderate ICC values. However, the model fit was poorer than the 2-component models (results not shown).

To classify the CpG sites into different reliability groups, Meng et al. [18] chose a cutoff of 0.5 for the posterior probability and suggested to exclude the “non-variable” sites in the low reliability group. In contrast, we set a rather small cutoff of 0.01 to include more sites with moderate ICC into the high reliability cluster, given the discussion above. In the ARIC study, the low reliability cluster contributes only ~4% of significant associations in the EWAS of smoking status, with only two associated sites having ICC < 0.1. We believe that validation using other approaches is especially important for sites with low ICC, for example, bisulphite pyrosequencing and replication in an external validation cohort. In this specific example, restricting analysis to CpG sites in the high reliability cluster would result in the loss of only a few significant association signals and could improve the power of EWAS by reducing the number of hypothesis tests by more than a half. To demonstrate, we calculated the statistical power at different effect sizes (%variance explained), based on a sample size of 2,500 and genome-wide significance level determined using Bonferroni correction. We assume 50% CpG sites will be excluded due to low ICC. We observe power increase of ~5% under this setting (in Additional file 1: Figure S5). For other studies, investigators may decide their own cutoff for the ICC. Even if all CpG sites on the chip are to be included in the association analysis, the ICC can still serve as a measure of how likely the association results are to be affected by technical errors.

Here, we aimed to describe the distribution of ICC using a statistical approach. Our results clearly approximate the observed distribution. However, it is unknown what biological or chemical factors explain the difference in performance of the corresponding probes on the chip between the low (including zero-ICC) and high reliability clusters. It is also unknown whether our results can be generalized to other populations or other types of tissues. The methylation profiling in ARIC samples are from white blood cells, which is a mixture of multiple cell types, and the participants are African Americans. It has been shown that both cell type composition [29] and sample ethnicity [22] can heavily influence DNA methylation pattern. It is unknown whether our results will still hold when a purified cell type, e.g., monocytes or neutrophils, or different tissue is used, or when samples from different ethnic groups are included. As an example, Etcheverry et al. [19] carried out an EWAS to identify CpG sites differentially methylated between glioblastoma and control samples using brain tissues. Among the 616 CpG sites identified, 584 are interrogated on the HM450 BeadChip. We examined the ICC values for these 584 sites in our data, and found 486 sites to belong to the high reliability group (ICC > 0.37), 88 to belong to the low reliability group, and 10 to belong to the zero-ICC group. Enrichment of significant associations in the high-ICC group in that study suggests that our results may have reasonable generality.

In addition, we used the “raw” methylation measures produced by the GenomeStudio software with minimal normalization. Other normalization procedures or use of M-values [30] instead of β-values may result in different ICC values. However, the same analytical approach used here can be applied in other studies, if technical replicates are included.

Another limitation of the study is that we did not take into account the estimation uncertainty when estimating the ICC, which can lead to different confidence intervals for this reliability measure. We will consider a weighted estimation approach in future studies, and re-classify the CpG sites on the HM450 chip.

## Conclusions

We examined the reliability of methylation measurements using the latest HM450 chip, and demonstrated that the CpG sites assayed on this array can be potentially classified according to different levels of reliability. We also evaluated the impact of measurement reliability on results of EWAS. The biological differences between the clusters of CpG sites need to be further investigated. The estimated ICC values for all CpG sites on the HM450 chip are available in Additional file 2. We hope that our results can provide additional guidance on inclusion/exclusion of CpG sites for future EWASs using the HM450 chip, and our analysis approach can be generalized to other types of methylation arrays.

## Methods

To evaluate the reliability of methylation measurement assayed on the HM450 chip, an intraclass correlation coefficient (ICC) can be calculated for each CpG site using the technical replicates. We aim to classify the CpG sites into multiple reliability groups, by modeling the distribution of ICC values using a mixture distribution.

### The ARIC methylation data

The Atherosclerosis Risk in Communities (ARIC) Study is a prospective cohort study of cardiovascular disease risk in four U.S. communities [31]. Between 1987 and 1989, 7,082 men and 8,710 women aged 45–64 years were recruited from Forsyth County, North Carolina; Jackson, Mississippi (African Americans only); suburban Minneapolis, Minnesota; and Washington County, Maryland. The ARIC Study protocol was approved by the institutional review board of each participating university. Participants underwent a baseline clinical examination (Visit 1) and four subsequent follow-up clinical exams (Visits 2–5). Written informed consent was obtained for each clinic exam, including that for genetic studies.

Genomic DNA was extracted from peripheral blood leukocyte samples using the Gentra Puregene Blood Kit (Qiagen) according to the manufacturer’s instructions (http://www.qiagen.com). Bisulphite conversion of 1 ug genomic DNA was performed using the EZ-96 DNA Methylation Kit (Deep Well Format) (Zymo Research) according to the manufacturer’s instructions (http://www.zymoresearch.com). Bisulphite conversion efficiency was determined by PCR amplification of the converted DNA before proceeding with methylation analyses on the Illumina platform using Zymo Research’s Universal Methylated Human DNA Standard and Control Primers.

Bisulphite-converted DNA from 2,905 African American participants at Visit 2 (1990–92; n = 2,504) or Visit 3 (1993–95; n = 441) were measured for methylation status using the HM450 chip. The degree of methylation is determined using Illumina GenomeStudio 2011.1, Methylation module 1.9.0 software. The methylation score for each CpG was represented as a beta (β) value calculated by dividing the fluorescence intensity of the methylated allele by the sum of the intensities of the methylated allele and unmethylated allele. β-values may take any value between 0 (non-methylated) and 1 (completely methylated). Background subtraction was conducted with the GenomeStudio software using built-in negative control bead types on the array. An average normalization was also applied in GenomeStudio to minimize scanner-to-scanner variation using ~90 normalization probe pairs included on the array which target housekeeping regions with no underlying CpG sites. These probes are used to independently calculate normalization values in the green and red channels so that all samples have the same average intensity.

Individuals (n = 32) were excluded from analysis if a pass rate for the DNA sample for the study participant was less than 99% (probes with a detection p-value >0.01/all probes on the array). Probes on the HM450 chip for which the pass rate was less than 99% (sample with a detection p-value >0.01 at probe/all samples) were not analyzed (n = 11,789).

Technical replicates were included for 130 samples (total n = 265 with 5 samples replicated 3 times), from which the ICC values are calculated for all probes. All except two of the replicate pairs were distributed on different chips.

### ICC estimation and modeling

At a specific CpG site, the methylation level can be modeled using a random-effects ANOVA model:


where *y*_*ij*_ is the measured methylation level for the *i*th replicate of biological sample (replicate set) *j*, *μ* the overall mean methylation at the site,  a random effect shared by all measures for sample *j* reflecting sample characteristics, and  a random noise term including technical errors for multiple measures of the same biological sample. *τ*_*j*_ and *ϵ*_*ij*_ are assumed to be uncorrelated. Here  presents variance between replicate sets and  represents variance within replicate sets. ICC is calculated as:


which takes values in [0,1] and measures the extent to which the measurements in a replicate-set resemble each other. An ICC of 1 indicates perfect measurement accuracy at the CpG site, and 0 implies little variation between independent samples but large measurement error between technical replicates. Thus, we can classify sites into clusters of varying degrees of reliability based on ICC values.

The ICC value can serve as a measure for the impact of batch effects on each probe. Consider a widely used random-effects model for batch effects:


where *l*(*ij*) indexes the batch (chip) where replicate *i* of sample *j* is located so *b*_*l*(*ij*)_ is the corresponding batch effect, and *X*_*jp*_ is the *p*th covariate (characteristic of biological sample), such as age or sex, for sample *j*. Comparing this model to the random-effects ANOVA model above, the within replicate set variation  is decomposed into variances of batch-specific measurement error (*b*_*l*(*ij*)_) and other technical error (*e*_*ij*_), and  corresponds to the between replicate set (biological sample) variation. When the measure of methylation is vulnerable to batch effects at a given CpG site, the variance of *b*_*l*(*ij*)_ is large, leading to small ICC.

Given that the ICC values are bounded in [0,1], especially taking into account the lower bound of 0, we fit a censored or truncated normal mixture models to the observed ICC values. For the censored normal mixture model, we assume that the CpG sites could be from two clusters: a low reliability cluster with ICC censored at 0, and a high reliability cluster with ICC distribution modeled by a normal distribution:


where *δ*_*i*_ = 1 for the censored observations (i.e., ICC = 0) and 0 otherwise, *p* is the mixing proportion for the first cluster,  the set of parameters, and *Φ*(⋅) and *ϕ*(⋅) denote the cumulative and probability distribution function of standard normal distribution, respectively.

Alternatively, to allow excess 0 s in the distribution of ICC, we separated CpG sites of ICC = 0 and modeled non-zero ICC values using a truncated normal and a normal mixture distribution:


The parameters in the mixture models were estimated using an Expectation-Maximization (EM) algorithm [32, 33]. For the purpose of comparison, we revised the log-likelihood function to include the CpG sites with ICC = 0:


where *p*_0_ and *n*_0_ denote the sample proportion and number of sites with ICC = 0, respectively, and *p*_1_ = *p*(1 - *p*_0_) and *p*_2_(1 - *p*)(1 - *p*_0_).

The two models were then compared using the maximized log likelihood values. The CpG sites interrogated by the HM450 chip can be classified based on the corresponding posterior probabilities, , computed from either of the two models.

### Data application: association with smoking status

To demonstrate how association patterns between methylation levels at CpG sites and phenotypic characteristics of individuals vary across the clustered sites, we investigated the cross-sectional association of methylation levels and smoking status in the ARIC Study. We fit a linear mixed-effects (LMM) model of methylation on self-reported smoking status (never/former smoker vs. current smoker), adjusting for age, sex, body mass index, self-reported alcohol consumption (never/former vs. current drinker), the top principal components of ancestry derived from genotype data, visit number, field center, and chip row position as fixed covariates. The values of the covariates were from the specific visit at which the participant’s sample was obtained. The batch (chip) effects were modeled as random effects. Probes with at least one single nucleotide polymorphism (SNP) within 50 base pairs of the CpG site and minor allele frequency (MAF) > .05 based on all samples in the 1000 Genomes project (http://www.1000genomes.org/) were excluded [22]. We used p < 10^-7^ as genome-wide significance based on a Bonferroni correction, and summarize the number of significant associations by the low and high reliability group, classified as above.

## Electronic supplementary material

Additional file 1: **Supplemental figures.** (DOCX 496 KB)

Additional file 2: **ICC values of all CpG sites.** (ZIP 8 MB)

## References

[1] Jones PA, Takai D (2001). The role of DNA methylation in mammalian epigenetics. Science.

[2] Robertson KD (2005). DNA methylation and human disease. Nat Rev Genet.

[3] Scarano MI, Strazzullo M, Matarazzo MR, D’Esposito M (2005). DNA methylation 40 years later: Its role in human health and disease. J Cell Physiol.

[4] Heyn H, Esteller M (2012). DNA methylation profiling in the clinic: applications and challenges. Nat Rev Genet.

[5] Qiu P, Zhang L (2012). Identification of markers associated with global changes in DNA methylation regulation in cancers. BMC Bioinformatics.

[6] Liu J, Chen J, Ehrlich S, Walton E, White T, Perrone-Bizzozero N, Bustillo J, Turner JA, Calhoun VD (2013). Methylation patterns in whole blood correlate with symptoms in schizophrenia patients. Schizophr Bull.

[7] Laird PW (2010). Principles and challenges of genomewide DNA methylation analysis. Nat Rev Genet.

[8] Sandoval J, Heyn H, Moran S, Serra-Musach J, Pujana MA, Bibikova M, Esteller M (2011). Validation of a DNA methylation microarray for 450,000 CpG sites in the human genome. Epigenetics.

[9] Marabita F, Almgren M, Lindholm ME, Ruhrmann S, Fagerstrom-Billai F, Jagodic M, Sundberg CJ, Ekstrom TJ, Teschendorff AE, Tegner J, Gomez-Cabrero D (2013). An evaluation of analysis pipelines for DNA methylation profiling using the illumina human Methylation450 BeadChip platform. Epigenetics.

[10] Dedeurwaerder S, Defrance M, Calonne E, Denis H, Sotiriou C, Fuks F (2011). Evaluation of the Infinium Methylation 450 K technology. Epigenomics.

[11] Touleimat N, Tost J (2012). Complete pipeline for Infinium((R)) Human Methylation 450 K BeadChip data processing using subset quantile normalization for accurate DNA methylation estimation. Epigenomics.

[12] Sun Z, Chai HS, Wu Y, White WM, Donkena KV, Klein CJ, Garovic VD, Therneau TM, Kocher JP (2011). Batch effect correction for genome-wide methylation data with Illumina Infinium platform. BMC Med Genet.

[13] Chen C, Grennan K, Badner J, Zhang D, Gershon E, Jin L, Liu C (2011). Removing batch effects in analysis of expression microarray data: an evaluation of six batch adjustment methods. PLoS One.

[14] Leek JT, Scharpf RB, Bravo HC, Simcha D, Langmead B, Johnson WE, Geman D, Baggerly K, Irizarry RA (2010). Tackling the widespread and critical impact of batch effects in high-throughput data. Nat Rev Genet.

[15] Leek JT, Johnson WE, Parker HS, Jaffe AE, Storey JD (2012). The sva package for removing batch effects and other unwanted variation in high-throughput experiments. Bioinformatics.

[16] Teschendorff AE, Zhuang J, Widschwendter M (2011). Independent surrogate variable analysis to deconvolve confounding factors in large-scale microarray profiling studies. Bioinformatics.

[17] Johnson WE, Li C, Rabinovic A (2007). Adjusting batch effects in microarray expression data using empirical Bayes methods. Biostatistics.

[18] Meng H, Joyce AR, Adkins DE, Basu P, Jia Y, Li G, Sengupta TK, Zedler BK, Murrelle EL, van den Oord EJ (2010). A statistical method for excluding non-variable CpG sites in high-throughput DNA methylation profiling. BMC Bioinformatics.

[19] Etcheverry A, Aubry M, de Tayrac M, Vauleon E, Boniface R, Guenot F, Saikali S, Hamlat A, Riffaud L, Menei P, Quillien V, Mosser J (2010). DNA methylation in glioblastoma: impact on gene expression and clinical outcome. BMC Genomics.

[20] Bibikova M, Le J, Barnes B, Saedinia-Melnyk S, Zhou L, Shen R, Gunderson KL (2009). Genome-wide DNA methylation profiling using Infinium(R) assay. Epigenomics.

[21] Chen YA, Lemire M, Choufani S, Butcher DT, Grafodatskaya D, Zanke BW, Gallinger S, Hudson TJ, Weksberg R (2013). Discovery of cross-reactive probes and polymorphic CpGs in the Illumina Infinium HumanMethylation450 microarray. Epigenetics.

[22] Barfield RT, Almli LM, Kilaru V, Smith AK, Mercer KB, Duncan R, Klengel T, Mehta D, Binder EB, Epstein MP, Ressler KJ, Conneely KN (2014). Accounting for population stratification in DNA methylation studies. Genet Epidemiol.

[23] Breitling LP, Yang R, Korn B, Burwinkel B, Brenner H (2011). Tobacco-smoking-related differential DNA methylation: 27 K discovery and replication. Am J Hum Genet.

[24] Wan ES, Qiu W, Baccarelli A, Carey VJ, Bacherman H, Rennard SI, Agusti A, Anderson W, Lomas DA, Demeo DL (2012). Cigarette smoking behaviors and time since quitting are associated with differential DNA methylation across the human genome. Hum Mol Genet.

[25] Joubert BR, Haberg SE, Nilsen RM, Wang X, Vollset SE, Murphy SK, Huang Z, Hoyo C, Midttun O, Cupul-Uicab LA, Ueland PM, Wu MC, Nystad W, Bell DA, Peddada SD, London SJ (2012). 450 K epigenome-wide scan identifies differential DNA methylation in newborns related to maternal smoking during pregnancy. Environ Health Perspect.

[26] Shenker NS, Polidoro S, van Veldhoven K, Sacerdote C, Ricceri F, Birrell MA, Belvisi MG, Brown R, Vineis P, Flanagan JM (2013). Epigenome-wide association study in the European Prospective Investigation into Cancer and nutrition (EPIC-Turin) identifies novel genetic loci associated with smoking. Hum Mol Genet.

[27] Monick MM, Beach SR, Plume J, Sears R, Gerrard M, Brody GH, Philibert RA (2012). Coordinated changes in AHRR methylation in lymphoblasts and pulmonary macrophages from smokers. American journal of medical genetics Part B, Neuropsychiatric genetics: the official publication of the International Society of Psychiatric Genetics.

[28] Bock C, Walter J, Paulsen M, Lengauer T (2008). Inter-individual variation of DNA methylation and its implications for large-scale epigenome mapping. Nucleic Acids Res.

[29] Jaffe AE, Irizarry RA (2014). Accounting for cellular heterogeneity is critical in epigenome-wide association studies. Genome Biol.

[30] Irizarry RA, Ladd-Acosta C, Carvalho B, Wu H, Brandenburg SA, Jeddeloh JA, Wen B, Feinberg AP (2008). Comprehensive high-throughput arrays for relative methylation (CHARM). Genome Res.

[31] The ARIC investigators (1989). The Atherosclerosis Risk in Communities (ARIC) Study: design and objectives. Am J Epidemiol.

[32] Dempster A, Laird N, Rubin D (1977). Maximum likelihood from incomplete data via the E-M algorithm. Journal of the Royal Statistical Society (B).

[33] Lee G, Scott C (2012). EM algorithms for multivariate Gaussian mixture models with truncated and censored data. Computational Statistics & Data Analysis.

