# Supplementary material for: Evaluation of microarray-based DNA methylation measurement using technical replicates: the Atherosclerosis Risk In Communities (ARIC) Study
Source: BMC Bioinformatics. 2014 Sep 19;15(1):312. doi: 10.1186/1471-2105-15-312 (PMC4180315; doi:10.1186/1471-2105-15-312)
Supplement: Supplementary file 1 — Additional file 1: Supplemental figures. (DOCX 496 KB) [file 12859_2014_6587_MOESM1_ESM.docx]

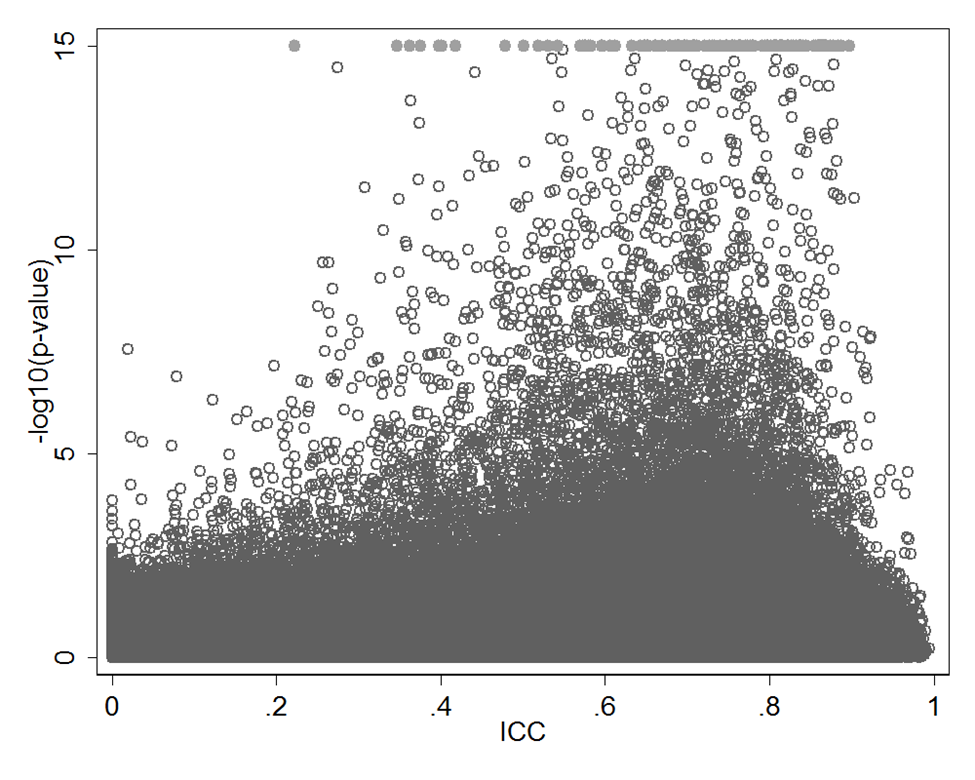


Supplemental Figure 1. Relationship between smoking-methylation association p-values and the ICC values for all sites on the HM450 chip. The –log_10_(p-value) are censored at 15 (solid circle).


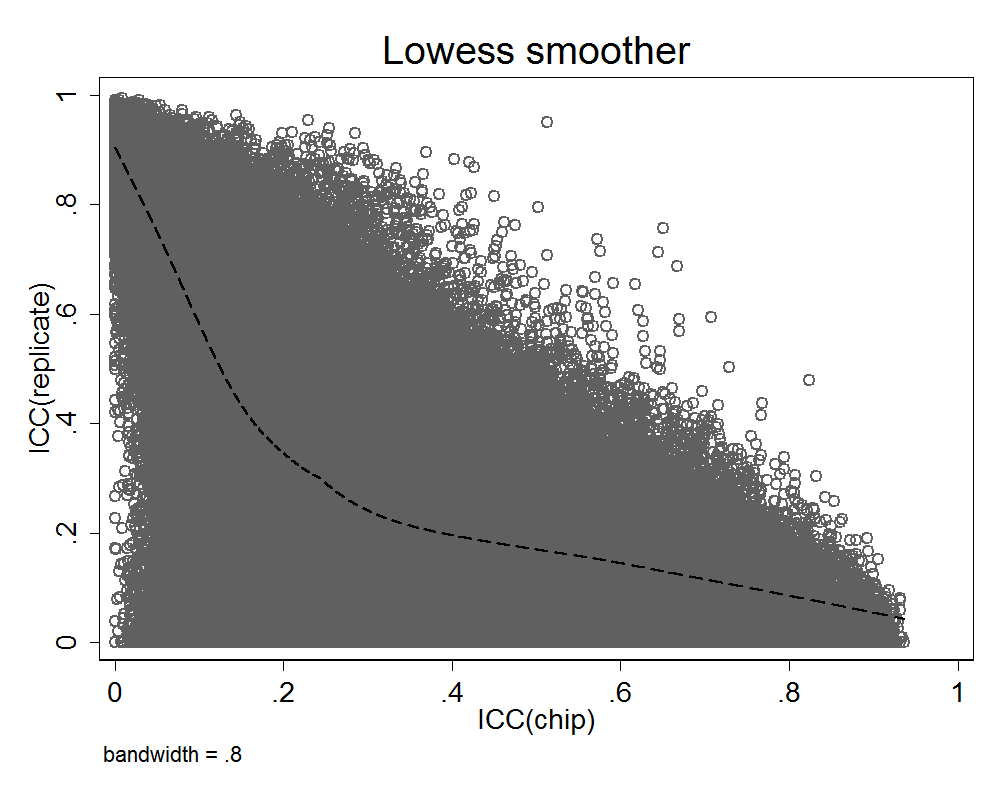


Supplemental Figure 2. The LOWESS fitted curve between ICC of replicates and ICC of chip effects.

(a)


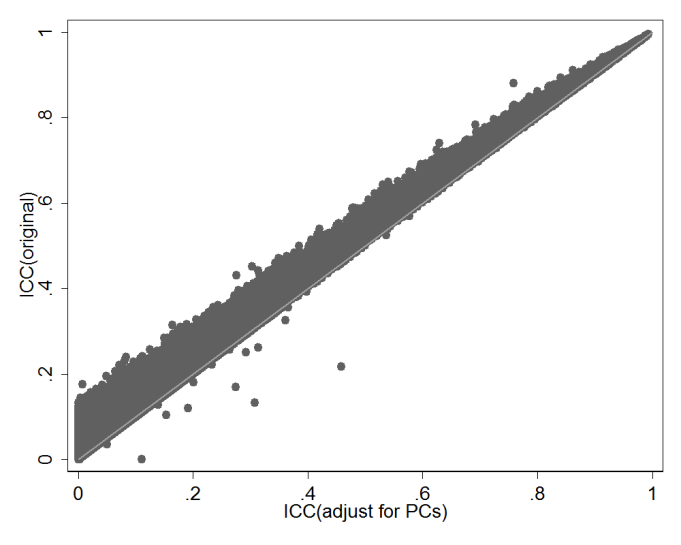


(b)


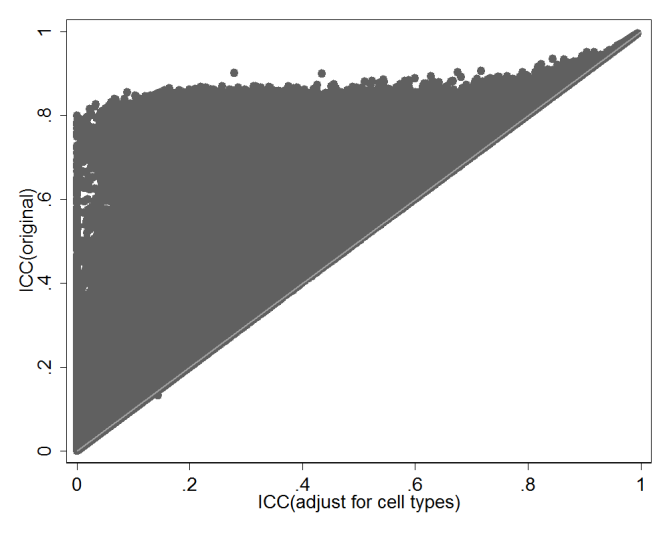


(c)


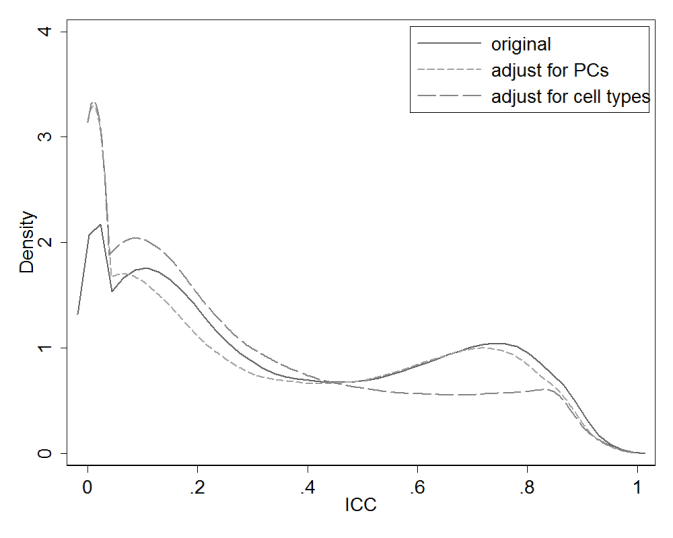


Supplemental Figure 3. Comparison of ICC with and without adjustment for covariates: (a) principal components (PCs) derived from genome-wide genotype data; (b) cell type composition estimated using Houseman et al.(2012) [[34](#_ENREF_34)]; (c) Distribution of ICC values without adjusting for covariate (solid), adjusting for PCs (medium dash), and adjusting for cell types (long dash).


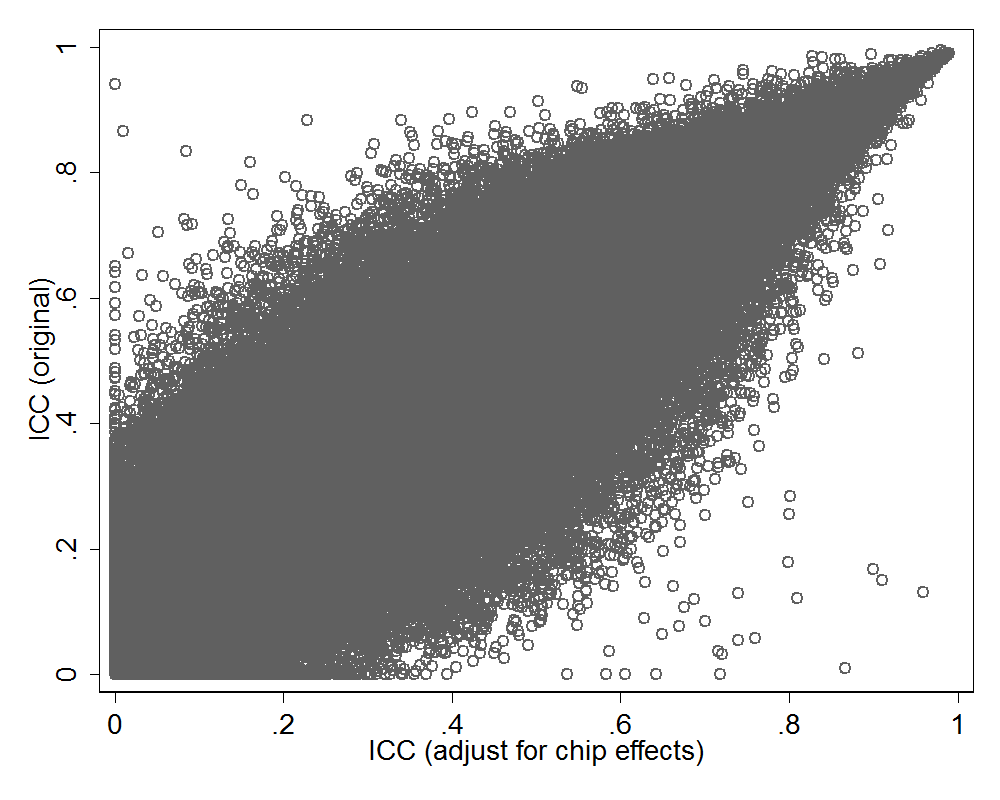


Supplemental Figure 4. Comparison of ICC with and without adjustment for chip effects.


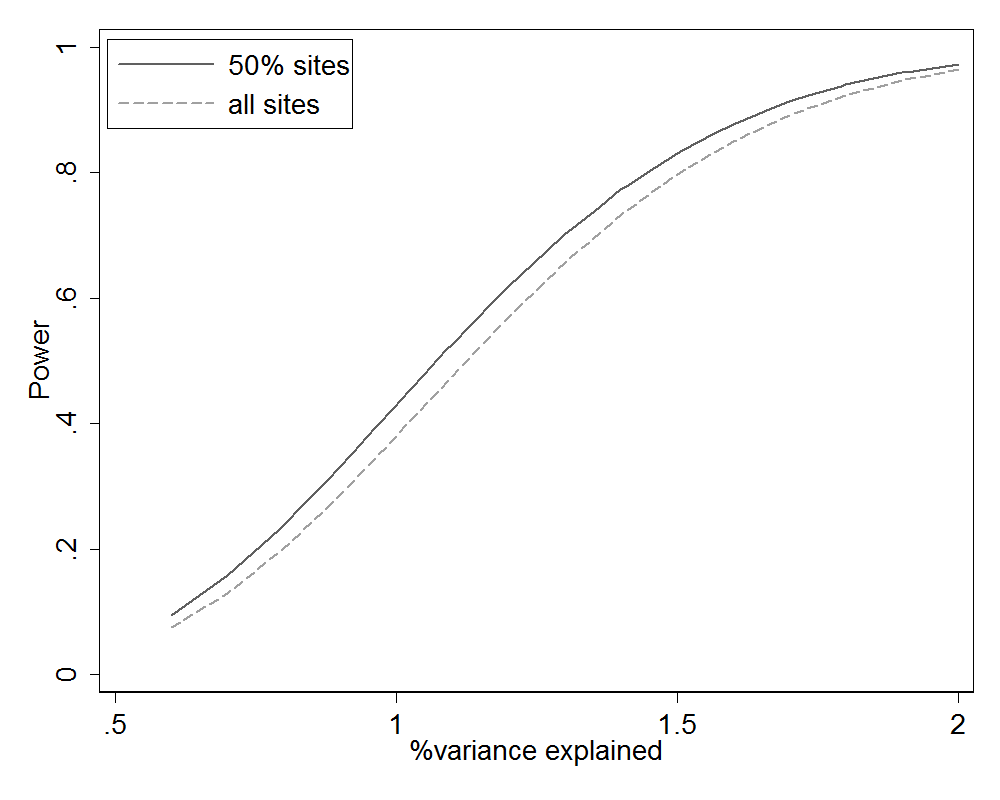


Supplemental Figure 5. Power to detect smoking-methylation association for different values of 100×R^2^ (%variance of methylation explained by the predictor). The calculation is based on a sample size of 2500 individuals, with genome-wide significance determined by Bonferroni correction: ≈1×10^-7^ when using all CpG sites; 2×10^-7^ when 50% CpG sites being excluded due to low ICC.
